# Mental health of medical workers in Japan during COVID-19: Relationships with loneliness, hope and self-compassion

**DOI:** 10.1007/s12144-021-01514-z

**Published:** 2021-02-20

**Authors:** Yasuhiro Kotera, Akihiko Ozaki, Hirotomo Miyatake, Chie Tsunetoshi, Yoshitaka Nishikawa, Tetsuya Tanimoto

**Affiliations:** 1 University of Derby, Derbyshire, Derby, UK; 2 Jyoban Hospital of Tokiwa Foundation, Iwaki, Fukushima Japan; 3 Medical Governance Research Institute, Minato-ku, Tokyo Japan; 4 Orange Home Care Clinic, Tawara, Fukui, Japan; 5 Kyoto University, Kyoto, Kyoto Japan

**Keywords:** Japan, Medical workers, Mental health, Loneliness, COVID-19

## Abstract

The current pandemic of the coronavirus disease 2019 (COVID-19) has negatively impacted medical workers’ mental health in many countries including Japan. Although research identified poor mental health of medical workers in COVID-19, protective factors for their mental health remain to be appraised. Accordingly, this study aimed to investigate relationships between mental health problems, loneliness, hope and self-compassion among Japanese medical workers, and compare with the general population. Online self-report measures regarding those four constructs were completed by 142 medical workers and 138 individuals in the general population. T-tests and multiple regression analysis were performed. Medical workers had higher levels of mental health problems and loneliness, and lower levels of hope and self-compassion than the general population. Loneliness was the strongest predictor of mental health problems in the medical workers. Findings suggest that Japanese medical workplaces may benefit from targeting workplace loneliness to prevent mental health problems among the medical staff.

## Introduction

Poor psychological status among medical workers can limit the quality and quantity of the medical workforce, leading to poor clinical outcomes of patients. This is particularly relevant to the current pandemic of the novel coronavirus disease 2019 (COVID-19), as it can cause negative impacts on mental health of medical workers (Matsuo et al., 2020; Moreno et al., 2020; Spoorthy et al., 2020). Considering strong stigma attached to mental illnesses among Japanese medical professionals (Ando, Yamaguchi, Aoki, & Thornicroft, 2013), directly engaging with mental health problems may not be effective as it can stimulate their stigma (Kotera et al., 2021). Previous studies elucidated the prevalence and levels of mental distress in medical workers during COVID-19 (Matsuo et al., 2020; Moreno et al., 2020; Spoorthy et al., 2020), however protective factors for their mental health were not evaluated. Healthful factors of mental health need to be appraised to identify effective interventions (Choi et al., 2020). Accordingly, we examined the relationships between mental health problems (depression and anxiety), loneliness (feeling alienated from others; Sekhon & Srivastava, 2019), hope (positive motivational construct, helpful during a crisis; Bernardo & Mendoza, 2020), and self-compassion (kindness towards oneself, associated with wellbeing; Sinclair, Kondejewski, Raffin-Bouchal, King-Shier, & Singh, 2017) during the COVID-19 pandemic in medical workers in Japan to appraise protective factors of mental health that are unique to this population, and suggest effective approaches.

## Methods

We aimed to contextualise the psychological status of medical workers by comparing with that of the general population in Japan. Online survey was distributed to Facebook groups formed by medical workers and the general population in June 2020, to which opportunity samples of 160 medical workers and 164 individuals in the general population agreed to participate. Participants had to (i) be 18 years old or older, (ii) be living in Japan at the time of the study, and (iii) have at least three years of experience living in Japan. To reduce the workload of participants, following four short scales were included in the survey: The Patient Health Questionnaire-4 (Löwe et al., 2010), Three-Item Loneliness Scale (Hughes, Waite, Hawkley, & Cacioppo, 2004), Adult State Hope Scale (Snyder et al., 1996), and Self-Compassion Scale-Short Form (Raes, Pommier, Neff, & Van Gucht, 2011). Of the agreed participants, 142 medical workers (28 doctors, 34 nurses, 29 pharmacists, 27 rehabilitation workers, and the remaining 24 included social workers and radiographers) and 138 individuals from the general population (85 full-time employees, 29 self-employed workers, 11 part-timers, and the remaining 13 included unemployed and homemakers) completed the survey (Table 1). Both groups satisfied the required sample size per as power calculation (119: Effect size f^2^ = 0.15, α = 0.05, Power = 0.95; (Faul, Erdfelder, Buchner, & Lang, 2009). Once data was screened for outliers and normalities, t-tests and multiple regression analysis were conducted. Ethical approval was obtained from the university research ethics committee. This study followed the Strengthening the Reporting of Observational Studies in Epidemiology (STROBE) reporting guideline.

**Table 1:** Participants characteristics and psychological variables

	Medical Workers (*n* = 142)		General Population (*n* = 138)	
	M	SD	M	SD
Characteristics				
Gender	F: 73% (*n* = 104), M = 27% (*n* = 38)		F: 61% (*n* = 84), M: 39% (*n* = 54)	
Age	39.90	12.10	46.39**	10.35
Job Role / Employment Status	Doctors	28	Full-Time Employees	85
	Nurses	34	Part-Timers	11
	Pharmacists	29	Self-Employed incl. Employers	29
	Rehabilitation Workers	27	Homemakers	5
	Social Workers	5	Unemployed	2
	Others incl. Nutritionists, Radiographers	19	Others incl. Students	6
Psychological Variables				
Mental Health Problems (measured by PHQ-4)	3.27**	2.78	2.37	2.31
Loneliness	4.73*	1.7	4.33	1.56
Hope	29.85	8.46	32.35**	7.82
Self-Compassion	3.13	0.58	3.34**	0.61

*p < .05, **p < .01 Significant difference between the two groups (noted on the higher value)

## Results

Our t-tests revealed that medical workers had high levels of mental health problems (*p* = 0.004, *t* = 2.88) and loneliness (*p* = 0.043, *t* = 2.04), and low levels of hope (*p* = 0.010, *t* = −2.62) and self-compassion (*p* = 0.004, *t* = −2.89), relative to the general population.

In multiple regression, first, gender and age were entered to adjust for their effects (Step 1), then, loneliness, hope and self-compassion scores were entered (Step 2). Multicollinearity was of no concern (Variance Inflation Factors <10). Loneliness (*p* < 0.001, β = 0.39), hope (*p* < 0.001, β = −0.28) and self-compassion (*p* = 0.007, β = −0.22) were significant predictors of mental health problems in medical workers, where loneliness was the strongest, and self-compassion was the weakest predictor; whereas hope was the strongest in the general population (*p* = 0.003, β = −0.27) (Table 2). The three predictors predicted a greater variance of mental health problems in medical workers than in the general population (44% > 30%).

**Table 2:** Multiple regression analysis for mental health problems

	Medical Workers					General Population				
	B	SE_B_	β	95.0% CI for B		B	SE_B_	β	95.0% CI for B	
Step 1				Lower	Upper				Lower	Upper
Gender	0.03	0.17	0.02	−0.31	0.37	−0.18	0.14	−0.11	−0.45	0.09
Age	−0.01	0.01	−0.13	−0.02	0.00	−0.01	0.01	−0.12	−0.02	0.00
Adj. R^2^	0.30%					1%				
Step 2										
Gender	0.09	0.13	0.05	−0.17	0.35	−0.11	0.12	−0.07	−0.34	0.12
Age	0.01	0.01	0.09	0.00	0.02	0.00	0.01	−0.03	−0.02	0.01
Loneliness	0.92	0.17	0.39***	0.58	1.25	0.50	0.25	0.20*	0.01	0.99
Hope	−0.31	0.08	−0.28***	−0.47	−0.14	−0.33	0.11	−0.27**	−0.54	−0.11
Self-Compassion	−1.17	0.42	−0.22**	−2.01	−0.33	−1.06	0.49	−0.21*	−2.04	−0.09
△ Adj. R^2^	44%					30%				

*Outcome variable* Mental Health Problems. *B* Unstandardized Coefficients, *SE*_*B*_ Standard Error of the Coefficient, *β* Standardized Coefficients. *p < 0.05; **p < 0.01; ***p < 0.001

## Discussion

During the COVID-19 pandemic in Japan, our analyses identified (i) poor mental health and weakened psychological resources of medical staff, and (ii) the importance of loneliness, hope and self-compassion to their mental health, particularly the strongest impact of loneliness. These findings suggest that the medical workforce in Japan can benefit from targeting loneliness in the workplace. As increasingly uncovered in occupational psychology, workplace loneliness is associated with limited job performance (Ozcelik & Barsade, 2018). Among the loneliness interventions, re-appraising maladaptive social cognitions of lonelier workers, conducted regularly, was found most effective (Masi, Chen, Hawkley, & Cacioppo, 2011), translating self-criticism into self-compassion. A caution is required as a concept of loneliness differs between individualistic and collective cultures, and these studies were primarily conducted in the Western individualistic contexts, different from the Japanese collectivism (Heu, van Zomeren, & Hansen, 2019). Moreover, our study used (a) self-report measures, susceptible to response biases (Kotera et al., 2020) and (b) a cross-sectional design, unable to elucidate causality of the variables—longitudinal studies should be conducted. Still, our findings would help identify practical interventions to be employed by Japanese medical teams during COVID-19. As the third wave has arrived in Japan in November 2020 (Kami, 2020), Japanese medical workers need to protect their mental health to continue to offer quality care for ever-increasing patients suffering from this fatal disease.

## Data Availability

The data that support the findings of this study are available from the corresponding author upon reasonable request.

## Code Availability

N/A
